# Editorial: “Is this a Dream?” – Evolutionary, Neurobiological and Psychopathological Perspectives on Lucid Dreaming

**DOI:** 10.3389/fpsyg.2021.635183

**Published:** 2021-02-09

**Authors:** Sergio A. Mota-Rolim, Katie M. de Almondes, Roumen Kirov

**Affiliations:** ^1^Brain Institute, Physiology and Behavior Department, and Onofre Lopes University Hospital, Federal University of Rio Grande do Norte, Natal, Brazil; ^2^Department of Psychology, Postgraduate Program in Psychobiology, Onofre Lopes University Hospital, Federal University of Rio Grande do Norte, Natal, Brazil; ^3^Institute of Neurobiology, Bulgarian Academy of Sciences, Sofia, Bulgaria

**Keywords:** lucid dreaming, dreams, consciousness, self-consciousness, REM sleep, nightmares and sleep quality, metacognition

Lucid dreaming (LD) is a peculiar state of dream consciousness occurring mostly during rapid eye movement (REM) sleep where individuals are aware of they are dreaming and even may control the oneiric content, while remaining asleep (Baird et al., 2019). Whereas this phenomenon has been described in many religions and by philosophers thousands of years ago (Van Eeden, 1913), scientific research on LD began last century (LaBerge et al., 1981). Recent epidemiologic studies demonstrate spontaneous LD to occur at least once in life in 51–55% of the human population, with their incidence being correlated positively with dream recall frequency and negatively with advancing age (Schredl and Erlacher, 2011; Saunders et al., 2016). Notably however, a higher incidence of LD is frequently associated with sleep disorders, psychiatric and neurological conditions, and also with elevated cognitive traits including meta-cognitive abilities and creativity (Blagrove and Hartnell, 2000; Blagrove and Pace-Schott, 2010; Filevich et al., 2015; Voss et al., 2018; Scarpelli et al., 2019; Siclari et al., 2020).

Since neurophysiologic and modern mindfulness-based techniques for induction of LD have been introduced (LaBerge et al., 1981; Tholey, 1988), it was proposed that practicing LD could boost cognitive and psychological functions, thus being implicated in treatment of psychiatric disorders and recurrent nightmares (Stumbrys et al., 2012; Mota-Rolim and Araujo, 2013). Indeed, neurophysiologic studies show that LD in REM sleep might provide the dreamers with a unique opportunity to navigate volitionally the oneiric content, possibly through induction of a sleep-wake-hybrid state (Voss et al., 2009) or by activating those brain structures and neural networks that underlie executive functions in wake and that are normally suppressed during sleep (Dresler et al., 2015; Baird et al., 2018). However, the actual psychological worth and neurobiological correlates of LD and their effects on daytime functioning still remain less well-understood.

In this Research Topic, we aimed to organize a discussion forum on current trends in LD research to foster future collaborations and enhance our understanding of LD and human consciousness. We welcomed seventeen submissions of which sixteen were published: six original research studies, two reviews, seven opinions, and one perspective article, which targeted different, yet partially overlapping aspects of LD research.

We begin with the historical review by Mota-Rolim et al. which offers a valuable summary of evidences from past cross-cultural religious sources that strongly support the view of LD as a natural feature of human conscious experience in sleep which influences the highest levels of conceptual thinking. The authors advocate for consideration of the accumulated in past empirical experiences with LD in contemporary research to further explore LD frequency and characteristics among modern non-religious societies. Investigating further the frequency and characteristics of LD in religious and non-religious communities could make a good sense in evaluating changes in locus of control (LOC), the degree to which people believe that they, as opposed to external forces, have control over the outcome of events in their lives (Rotter, 1966) in association with dreaming and with LD, in particular (Blagrove and Hartnell, 2000). A recent study demonstrated high prevalence of supernatural agent in dream imagery among Hindu Nepalese informants (Nordin and Bjälkebring, 2019).

In contrast, studies on LD in non-religious participants suggest LD characteristics to be associated with higher cognitive and memory functions including meta-cognitive abilities and creativity and also with various personality traits (Blagrove and Pace-Schott, 2010). Two original studies published in our topic clearly show such interesting associations. Firstly, the large community-based study in Chinese students demonstrates significantly lower bizarreness density (BD) in subjects with LD than in participants without LD, while meta-cognition traits (self-reflection and insight) are negatively associated with BD in both LD and non-LD (Yu and Shen). Secondly, the online-based survey among 455 English responders who had previously experienced LD points to complex relationships across LD characteristics (frequency and extent of sustained awareness and control), REM sleep dissociative states (sleep paralysis and nightmares), proneness to reality deficits, and paranormal experiences and beliefs. The study shows paranormal experiences to correlate positively with LD features and parasomnia-related dissociative states, while paranormal beliefs are only associated with sleep paralysis and nightmares (Drinkwater et al.). Collectively, these two studies infer, firstly, LD to be associated with less bizarreness and higher meta-cognition in a state-dependent manner, and secondly, proneness to reality deficits to be associated with LD and sleep dissociative states trait-dependently.

Next, the opinion of Drinkwater et al. discuss possibilities for socially aversive traits, machiavellianism, narcissism and psychopathy, known as dark triad to affect dreams and LD features. They analyze the studies available and highlight substantial research limitations pointing to the need of future investigations in this area. Further, the opinion of Horton provides interesting and meaningful information about the role of LD for emotional processing, while discussing correlates of LD incidence with various psychopathologies. Definitions of key concepts in research on LD and non-LD regarding cognition, control of dream content, and conscious states are deeply emphasized.

Some contributions consider the current applicability, benefits and limitations of cultivating LD. The opinion of Vallat and Ruby rises serious concerns that LD and training procedures to increase their frequency may be harmful to the normal sleep and daytime functioning, while impacting negatively on sleep regulatory mechanisms. The authors argue, firstly, that methodologies used for LD induction alter sleep integrity, and secondly, that brain state during LD is neither that of wake, nor that of REM sleep but is rather a hybrid state which is naturally infrequent or unlikely. In the same line of discussion, the opinion of Soffer-Dudek outlines some potential benefits from LD, while considering risks at fragmented sleep that deliberate induction of LD may produce, which adverse effects are frequently disregarded. The author argues that continuous deliberate LD induction also may produce detrimental psychotic and dissociative mental sates through blurring of boundaries between reality and dreaming. The opinion of Mota-Rolim provides discussion on whether it is physiologically possible to move the eyes consciously and voluntarily during a pure REM sleep episode, as required for the pre-agreed eye movements (PAEM) technique, which is used to objectively indicate a LD. Results that gave rise to the “scanning hypothesis” were critically reviewed. The author concludes that since the PAEM constitutes the most used method to scientifically study LD, a consensus on how to apply this technique in a standardized way is still clearly warranted.

The original research by Ribeiro et al. describes and compares dream experience frequencies (dream, lucid dreams, awareness, and control) in association with sleep quality among students and in a general population sample. It is found that the frequency of all dream experiences could not predict negative impact on the quality of sleep. Aspy conducted an original research among 355 participants to evaluate the effectiveness of five different techniques for induction of LD. Major findings indicated that all techniques were effective regardless of baseline LD frequency or prior experience with LD. No adverse effects on sleep quality were found. Erlacher and Stumbrys conducted an insightful sleep laboratory controlled experiment using four different wake-up-back-to-bed (WBTB) conditions and a mnemonic technique (MILD) to explore reliably the effectiveness of this technique. The overall pattern of obtained results shows that through applying a combination of WBTB and MILD, detectable LD can be effectively induced in people who are not selected for their LD abilities. Regarding the neurophysiological approaches for inducing LD, the opinion of Mota-Rolim et al. describes portable devices for induction of LD, their scientific backgrounds and their reliability. The authors found that there are 10 portable devices in LD induction technologies, but only one has been empirically tested with published results and two provided minimal technical information on how their algorithm detects REM sleep online. In addition, association of the portable devices with cognitive and pharmacological techniques and their potential to improve the reliability of LD induction high-technologies were considered.

Regarding the clinical applicability of LD therapy (LDT), the manuscript of Macêdo et al. reviews existing literature of the effectiveness of LDT for treatment of nightmares. The authors conclude that although induction of LD may be a feasible aid in the treatment of patients with nightmares through minimizing their frequency, intensity and psychological distress, the available studies are still scarce and do not provide consistent results. Several study limitations should be considered in future clinical trials. Similarly, the case-controlled study by Holzinger et al. investigates the effectiveness LDT for coping with nightmares and sleep problems in patients suffering from posttraumatic stress disorder (PTSD). The authors found no effect of the LDT on the investigated sleep variables and no a correlation between reduction of nightmare severity and changes in PTSD-profile. However, levels of anxiety and depression decreased significantly in the course of therapy. The opinion of van Heugten-van der Kloet and Lynn discuss the enhanced insight and meta-consciousness through LD as possible psychological approach in coping with dissociation and psychotic illnesses, in order to reduce negative emotions in patients suffering from psychiatric disorders. They also acknowledge the high costs and the frequently observed ineffectiveness of this approach and advocate for opening novel research programs focusing on the relations among dissociation, the sense of self, and sleep and dreaming.

Finally, the perspective study by Holzinger and Mayer describes and models neurophysiological evidence for the seven awareness criteria of LD proposed by Tholey (1988). Each of the awareness criteria was analyzed separately with regard to its underlying neural circuits. It is hypothesized that not one, but several regions are involved in the state of lucid dreaming. Altogether, these contributions provide important psychological, neurophysiological, methodological, and clinical implications in future LD research.

## Author Contributions

SM-R, KA, and RK drafted the manuscript and are accountable for all aspects of the work.

## Conflict of Interest

The authors declare that the research was conducted in the absence of any commercial or financial relationships that could be construed as a potential conflict of interest.
